# Masseter Muscle Morphometric Changes After Botulinum Toxin Type A Injection: A Prospective 3-Month Study

**DOI:** 10.3390/toxins18070306

**Published:** 2026-07-15

**Authors:** Matteo Val, Ludovica Zucchini, Matteo Pollis, Luca Guarda-Nardini, Luca Lombardo, Daniele Manfredini, Anna Colonna

**Affiliations:** 1 Unit of Oral and Maxillofacial Surgery, Ca’ Foncello Hospital, 31100 Treviso, Italy; luca.guarda@unipd.it; 2 Department of Medical Biotechnologies, University of Siena, 53100 Siena, Italy; matteo.pollis@gmail.com (M.P.); dr.annacolonna@gmail.com (A.C.); 3 Postgraduate School of Orthodontics, University of Ferrara, 44121 Ferrara, Italy; ludovica.zucchini@edu.unife.it (L.Z.); luca.lombardo@unife.it (L.L.)

**Keywords:** botulinum toxin, myofascial pain, bruxism, temporomandibular disorders, 3D facial morphometry, structured-light scanning, masseter muscle

## Abstract

Myofascial pain of the masticatory muscles, frequently associated with bruxism, is a prevalent condition with substantial impact on quality of life. Botulinum toxin type A (BoNT-A) is used as an adjunctive treatment for refractory cases, but the temporal pattern of masseter morphometric change after injection remained incompletely characterised. This prospective observational pilot study was designed and reported strictly as a 3D imaging and morphometric methodology study; clinical pain and patient-reported outcomes were outside its scope and were not assessed. Eleven adults with myofascial pain and bruxism received BoNT-A (100 U total: 30 U per masseter side, 20 U per anterior temporalis side). Structured-light 3D facial scans (EinScan H, Shining 3D) were acquired at baseline (T0), one month (T1), and three months (T2). A standardised masseter region of interest (“TGL area”) was defined using cutaneous landmarks (tragus, gonion, labial commissure). Measurement precision was confirmed by intra-session repeatability (0.083 ± 0.020 mm), registration accuracy (RMS error 0.189–0.200 mm), and excellent landmark reliability (mean ICC [3, 1] = 0.955, 95% CI: 0.843–0.991). Repeated-measures ANOVA showed a significant effect of time on bilateral surface displacement (F(1.85,18.52) = 14.39, *p* < 0.001, η^2^G = 0.44), with significant reductions at T1 (–0.716 ± 0.480 mm; *p* = 0.002, Hedges’ g = 2.03) and T2 (–0.847 ± 0.588 mm; *p* = 0.002, g = 1.96) relative to baseline, and no significant change between T1 and T2 (*p* = 1.000). Changes were bilaterally symmetric (symmetry index at T1: 0.231 ± 0.154 mm). These findings indicate that 3D structured-light scanning reliably detects subtle facial contour changes after BoNT-A injection and may complement clinical and patient-reported assessments; the pilot design and small sample preclude definitive conclusions.

## 1. Introduction

Myofascial pain of the masticatory muscles represents one of the most prevalent subtypes of temporomandibular disorders (TMDs) and constitutes a major source of orofacial pain with relevant impact on quality of life [1,2,3]. The condition is characterised by localised or regional muscle pain, often accompanied by functional limitation and muscle fatigue [4]. Bruxism—defined as repetitive jaw-muscle activity involving teeth clenching, grinding, or bracing—is a frequently observed comorbidity, potentially perpetuating muscle hyperactivity and pain [5,6].

First-line management is founded on conservative and reversible approaches, including patient education, behavioural modification, physical therapy, occlusal appliances, and pharmacological support [7,8]. A subset of patients develops persistent or treatment-refractory myofascial pain despite adequate conservative care, necessitating consideration of adjunctive interventions.

Botulinum neurotoxin type A (BoNT-A) has been proposed as an adjunctive therapeutic option for selected patients with masticatory myofascial pain and bruxism [9,10,11]. The agent acts through irreversible cleavage of SNAP-25 at the neuromuscular junction, blocking acetylcholine release and producing reversible chemodenervation [12,13,14,15]. In addition to reducing muscular contractility, BoNT-A induces temporally defined structural changes in treated muscles, including volumetric reduction and architectural remodelling [16,17,18]. Clinical studies have documented analgesic and functional benefits of BoNT-A in myofascial pain cohorts [9,19,20], and recent perspectives discuss its potential integration into multidisciplinary TMD management protocols [20,21].

Despite growing clinical interest, the temporal dynamics and magnitude of morphometric changes in the masseter region following BoNT-A injection in patients with symptomatic myofascial pain remain incompletely characterised. Most prior investigations focused on aesthetic applications in asymptomatic individuals or on CT-based volumetric assessment in heterogeneous cohorts, limiting their generalisability to pain-focused clinical populations [22,23,24]. Longitudinal 3D surface imaging studies specifically examining symptomatic patients, using standardised ROI methodology, are scarce.

Three-dimensional (3D) facial imaging technologies based on structured light provide non-invasive, radiation-free alternatives to volumetric assessment [25,26,27]. These methods enable detection of sub-millimetric surface displacements and offer complementary information to clinical and patient-reported outcomes [26,27].

This prospective observational pilot study aimed to evaluate the magnitude and temporal pattern of 3D morphometric changes in the masseter region following BoNT-A injection in adults with myofascial pain and concurrent bruxism and to report measurement reliability indices for the imaging methodology employed. The primary outcome was mean signed surface displacement within the standardised masseter ROI at T1 and T2 relative to baseline. Secondary outcomes included displacement bin distributions, temporal recovery patterns, and bilateral symmetry of changes. Given the pilot nature of the study, results are intended to be hypothesis-generating rather than confirmatory. Consistent with this methodological focus, the study did not evaluate clinical pain, functional limitation, or patient-reported outcomes, which fall outside its scope; readers seeking evidence on the clinical efficacy of BoNT-A for myofascial pain are directed to the clinical trial literature discussed in Section 3.4.

## 2. Results

### 2.1. Study Population and Demographics

Of 13 participants initially screened, two were unable to complete follow-up assessments due to scheduling conflicts unrelated to adverse events, yielding a final cohort of 11 participants (1 male, 10 female; age range 28–75 years; mean age 50.3 ± 15.1 years). All 11 enrolled participants completed baseline, one-month, and three-month imaging assessments without scan repetition. Demographic and baseline characteristics are summarised in Table 1. All participants met DC/TMD criteria for myofascial pain and definite bruxism per the STAB questionnaire. No serious adverse events were reported; localised ecchymosis and mild transient oedema at injection sites were observed in 3 participants (27.3%) and resolved within 5–7 days.

### 2.2. Imaging Reliability and Measurement Precision

Intra-session repeatability. Mean surface deviation between paired T0 scans (brief repositioning, same session) was 0.083 ± 0.020 mm (range: 0.050–0.120 mm; 95th percentile: 0.112 mm) across the 11 participant pairs. All values were well below the a priori noise tolerance threshold of ±0.5 mm, confirming that acquisition-related noise represents a negligible contribution to the measured longitudinal displacement signal.

Registration accuracy (RMS error). Mean RMS registration errors were: T0-T1, 0.189 ± 0.028 mm (range: 0.140–0.230 mm); T0-T2, 0.200 ± 0.031 mm (range: 0.150–0.250 mm); T1-T2, 0.172 ± 0.026 mm (range: 0.130–0.220 mm). All RMS values were substantially below the ±0.5 mm noise tolerance band, supporting the adequacy of the ICP registration pipeline for detecting the observed morphometric changes.

Landmark reliability. ICC [3, 1] values for TGL landmark coordinates on the retested subset of four scans are summarised in Table 2. All ICC values exceeded 0.93, with a mean ICC across all landmarks and coordinates of 0.955 (95% CI: 0.843–0.991), indicating excellent intra-rater reliability for ROI definition.

### 2.3. Primary Outcome: Mean Surface Displacement

Mean bilateral surface displacement values are summarised in Table 3. Repeated-measures ANOVA demonstrated a statistically significant main effect of time (F(1.85, 18.52) = 14.39, *p* < 0.001, η^2^G = 0.44, Greenhouse-Geisser ε = 0.926), indicating that surface displacement differed significantly across the three timepoints.

Bonferroni-corrected post hoc comparisons revealed significant reductions from baseline at both T1 (mean change: −0.716 ± 0.480 mm, 95% CI: −1.000 to −0.433 mm; t(10) = 4.95, *p* = 0.002, Hedges’ g = 2.03) and T2 (mean change: −0.847 ± 0.588 mm, 95% CI: −1.195 to −0.500 mm; t(10) = 4.78, *p* = 0.002, Hedges’ g = 1.96). The difference between T1 and T2 was not statistically significant (*p* = 1.000, Hedges’ g = 0.23), indicating that displacement magnitude did not decrease appreciably between one and three months. Unilateral analyses confirmed the same directional pattern on both sides (Table 3).

### 2.4. Secondary Outcome: Displacement Bin Distributions

Across all timepoints and analyses, the majority of surface points (approximately 60–75%) remained within the minimal displacement range (0 to ±1 mm), reflecting the absence of gross facial contour changes. However, a progressive shift toward negative displacement values was evident following BoNT-A injection.

T0→T1 comparison. At one month, a statistically significant redistribution of surface points was detected in the 0 to +1 mm range (bilateral: Wilcoxon W = 1.0, *p* = 0.002, r = 0.93), reflecting a shift toward negative displacement. The −1 to −2 mm range showed a significant increase in bilateral analysis (W = 3.0, *p* = 0.005, r = 0.85), and the left unilateral −1 to −2 mm bin likewise reached significance (W = 2.0, *p* = 0.003, r = 0.90). The −2 to −3 mm range did not reach significance at this timepoint (*p* > 0.10).

T0→T2 comparison. The cumulative three-month comparison demonstrated the greatest overall shift toward negative values. The −1 to −2 mm range showed a highly significant increase (bilateral: W = 0.0, *p* = 0.001, r = 0.99), and the −2 to −3 mm range also reached significance (W = 6.0, *p* = 0.014, r = 0.74), consistent with persistent structural modification at three months.

T1→T2 comparison. Between one and three months, no bin-level comparison reached statistical significance (all *p* > 0.05), consistent with the primary outcome showing no significant change in mean displacement between these timepoints. This pattern may reflect either a plateau in the morphological response or insufficient statistical power to detect smaller bin-level changes within this pilot sample.

Key bin-level findings are summarised in Table 4.

### 2.5. Bilateral Symmetry

Morphometric changes were broadly symmetric between the left and right sides at both follow-up timepoints. The bilateral symmetry index (mean absolute difference between left and right ROI mean displacement) was 0.231 ± 0.154 mm at T1 and 0.565 ± 0.896 mm at T2. Wilcoxon signed-rank tests comparing left and right mean displacement found no significant asymmetry at T1 (W = 27.0, *p* = 0.638) or T2 (W = 23.0, *p* = 0.400), supporting bilateral symmetry of the morphometric response. The larger symmetry index at T2 reflects inter-individual variability in recovery trajectory rather than a systematic directional asymmetry.

### 2.6. Temporal Trajectory Summary

Synthesis across primary and secondary outcomes reveals a consistent temporal pattern: (i) at one month (T0→T1), significant bilateral volumetric reduction in the masseter region, consistent with pharmacologic peak effect; (ii) between one and three months (T1→T2), no statistically significant change in mean displacement or bin distributions, suggesting a morphological plateau within this window; (iii) at three months (T0→T2), sustained net volumetric reduction relative to baseline, with statistical significance in both the −1 to −2 mm and −2 to −3 mm displacement ranges, indicating incomplete morphological recovery within the observation period. This pattern is consistent with the known pharmacokinetics of BoNT-A and with structural muscle changes that may outlast maximal functional effects.

## 3. Discussion

Taken together, these findings position 3D structured-light scanning as a measurement tool precise enough to capture a real, reproducible masseter contour change after BoNT-A injection and temporally coherent with the toxin’s known pharmacodynamics: a marked reduction emerging by one month, essentially unchanged at three months, and symmetric across sides. Rather than reading the technical, mechanistic, comparative, and clinical observations below as isolated segments, we interpret them as facets of a single process—a structural correlate of chemodenervation that non-invasive imaging can now track directly. This integrated reading, together with the methodological limitations discussed in Section 3.5, is intended to guide how the imaging approach itself, rather than the BoNT-A treatment, should be positioned in future confirmatory research.

### 3.1. Three-Dimensional Surface Imaging as a Morphometric Tool

Three-dimensional surface imaging technologies, particularly structured-light scanners, offer several practical advantages over conventional 2D photography and CT-based volumetry: non-invasive acquisition, absence of ionising radiation, sub-millimetric spatial resolution, and standardised imaging protocols [25,26,27]. These characteristics make them potentially suitable for longitudinal morphometric monitoring in clinical research settings. The measurement reliability data reported here—intra-session repeatability of 0.083 mm, RMS registration errors below 0.21 mm, and ICC values exceeding 0.93—are encouraging and suggest that the observed displacement signal (mean bilateral change: −0.72 mm at T1) is substantially larger than measurement noise. However, these reliability data were obtained from a small subset of scans (*n* = 4 for ICC), and independent validation in larger samples would be required before making strong claims about the method’s general performance.

The standardised ROI approach (“TGL area” defined by tragus, gonion, and labial commissure) provides a reproducible framework applicable across participants, though the manual landmarking process introduces an inherent variability component that the ICC data only partially capture. Automated or semi-automated landmark detection approaches could reduce this source of variability in future studies.

### 3.2. Botulinum Toxin Type A: Mechanism and Temporal Pattern

BoNT-A exerts its effect through irreversible cleavage of SNAP-25 at the neuromuscular junction, blocking acetylcholine release and producing reversible chemodenervation [12,13,14,15]. The clinical timeline of BoNT-A effects follows a characteristic trajectory: onset at 3–5 days, peak effect at 4–6 weeks, and gradual recovery over 12–16 weeks as neuromuscular repair and collateral reinnervation occur [28,29].

Beyond functional effects, BoNT-A induces structural changes in treated muscles, including fibre atrophy and architectural remodelling [30,31]. These structural adaptations may persist longer than peak pharmacologic effect, and imaging studies have documented incomplete morphological recovery even at 12 weeks [11,32], and functional studies combining electromyography and bite force assessment have similarly documented prolonged effects on neuromuscular function [33,34,35,36,37]. Our findings—a significant reduction at T1 with no significant further change between T1 and T2—are broadly consistent with this trajectory, though the absence of data beyond three months limits conclusions about the full recovery pattern.

### 3.3. Comparison with Prior Morphometric Studies

The morphometric direction and approximate magnitude of changes observed here are broadly consistent with prior investigations of BoNT-A-induced masseter changes. Lee et al. [22] employed 3D laser scanning to assess lower facial contouring following BoNT-A injections and documented volume reduction peaks within 4 weeks with persistence through 12 weeks. Shim et al. [23] similarly reported measurable masseter volume reduction at approximately 4 weeks. Yu et al. [38] described a prospective series assessing lower facial contouring changes following BoNT-A injection, similarly documenting contour reduction at 4–6 weeks. Zhang et al. [39] used 3D reconstruction to characterise masseter morphology following BoNT-A and documented early changes (4–6 weeks) with gradual recovery.

However, these prior studies predominantly enrolled aesthetic patients without myofascial pain, and direct quantitative comparisons are limited by methodological differences in imaging technique, ROI definition, and outcome metrics. Whether the morphometric response differs between aesthetic and symptomatic populations—whether due to differences in baseline muscle mass, bruxism-related hypertrophy, or clinical context—remains an open question that requires adequately powered comparative studies.

### 3.4. Relationship to Clinical Evidence

The present morphometric study does not directly assess clinical pain or functional outcomes. In patients with myofascial pain and bruxism, the clinical rationale for BoNT-A is supported by evidence of analgesic and functional benefit in selected cohorts [9,19,20], though the literature remains heterogeneous in terms of patient selection, outcome measures, and dosing protocols, as reflected by variable reported efficacy of low-dose regimens for bruxism-related chronic pain [40,41]. Alternative minimally invasive approaches for masticatory myofascial pain, including masseteric nerve block, trigger point injections, and dry needling, have also been compared with BoNT-A in the literature [42]. Recent reviews have underscored the importance of positioning BoNT-A as an adjunctive intervention within comprehensive multidisciplinary TMD management, rather than a standalone treatment [20,21]. The structural changes documented here may provide a complementary perspective on the biological mechanisms underlying clinical response, though establishing a direct relationship between morphometric change and clinical improvement would require simultaneous assessment of both endpoints in future studies.

### 3.5. Limitations

Several limitations must be acknowledged. First, the sample size (*n* = 11) is small, and the study was explicitly designed as a pilot investigation; statistical power is limited, and results should be interpreted as preliminary. Second, the absence of a control group precludes definitive attribution of observed morphometric changes to BoNT-A versus natural temporal variation or regression to the mean. Third, follow-up was limited to three months, which is insufficient to characterise the full recovery trajectory or to inform retreatment intervals. Fourth, injection of the temporalis muscle in addition to the masseter complicates attribution of masseter-specific morphometric changes. Fifth, the landmark reliability data were obtained from only four scans, limiting their precision. Sixth, no formal assessment of pain, functional improvement, or quality-of-life outcomes was included; this was a deliberate scope choice, as the study was designed as an imaging and morphometric methodology pilot rather than a clinical efficacy trial. Seventh, bite tension was standardised through verbal cueing rather than a mechanical interocclusal device; while this avoided soft-tissue displacement artefacts at the ROI, it introduced a potential source of inter-timepoint variability that a calibrated bite-registration approach could reduce in future studies. Finally, the predominantly female cohort may limit generalisability. Cross-validation of the structured-light scanning technique against an ionising-radiation-based modality such as CBCT was not undertaken: this soft-tissue surface-imaging approach is already established in the BoNT-A morphometric literature [22,23,27,38,39], and exposing participants to an additional radiographic acquisition with no direct diagnostic benefit, purely for methodological cross-validation, would not have met the risk–benefit threshold required for ethics committee approval.

Notable strengths include the prospective design, standardised longitudinal assessment at three timepoints, reporting of measurement reliability indices, blinded landmark placement, and focus on a symptomatic myofascial pain population that has been underrepresented in prior morphometric studies.

### 3.6. Future Directions

Future studies should address these limitations through larger sample sizes with formal a priori power calculations, inclusion of control groups (e.g., sham injection or wait-list), longer follow-up periods (≥8–9 months) to characterise full morphological recovery, and simultaneous assessment of clinical endpoints (pain intensity, functional limitation, quality of life). Integration of morphometric data with electromyographic and force measurements would further elucidate the relationship between structural and functional changes. Independent validation of the imaging methodology in larger cohorts and exploration of automated landmark detection approaches would strengthen the reliability framework.

## 4. Conclusions

This prospective pilot study provides preliminary 3D morphometric data on masseter soft tissue changes following BoNT-A injection in adults with myofascial pain and bruxism. A statistically significant bilateral volumetric reduction was observed at one month (mean displacement: −0.72 mm, Hedges’ g = 2.03), with sustained change at three months and no significant further displacement between the two follow-up timepoints. Measurement reliability indices were satisfactory within the constraints of the pilot design.

Structured-light 3D surface imaging demonstrated sufficient precision to detect the observed morphometric signal and merits further investigation as a non-invasive, radiation-free complement to clinical assessments in this context. The findings are hypothesis-generating and should be interpreted cautiously given the sample size, absence of a control group, and limited follow-up duration. Larger prospective studies with concurrent clinical endpoints, control groups, and extended follow-up are needed to confirm and extend these observations.

## 5. Materials and Methods

### 5.1. Study Design, Setting, and Participants

This prospective observational pilot study was conducted between March and October 2025 at the Maxillofacial Surgery Unit of Ca’ Foncello Hospital, Treviso, Italy. Adult patients with myofascial pain of the masticatory muscles were consecutively recruited from the surgical clinic population.

Inclusion criteria were: (i) age ≥ 18 years; (ii) diagnosis of myofascial pain of the masticatory muscles according to DC/TMD criteria (pain on palpation of ≥3 sites among 20 predefined palpation points) [4]; (iii) presence of bruxism according to the Italian-validated version of the Standardised Tool for the Assessment of Bruxism (STAB) [5]; and (iv) willingness to undergo BoNT-A treatment and complete all follow-up imaging.

Exclusion criteria included: neurological, psychiatric, or rheumatologic disorders affecting neuromuscular function; pregnancy or breastfeeding; craniofacial syndromes; medications known to impair neuromuscular transmission; prior facial surgery or cosmetic procedures; previous BoNT-A injections within 12 months; diagnosis of articular, neuropathic, vascular, or primary headache-related orofacial pain; and inability to complete follow-up assessments.

The study was conducted in accordance with the Declaration of Helsinki and approved by the local Ethics Committee (Approval ID: 581/CE Marca, Treviso, Italy). All participants provided written informed consent prior to enrolment. Reporting followed the STROBE guidelines for observational studies.

**Figure 1:**
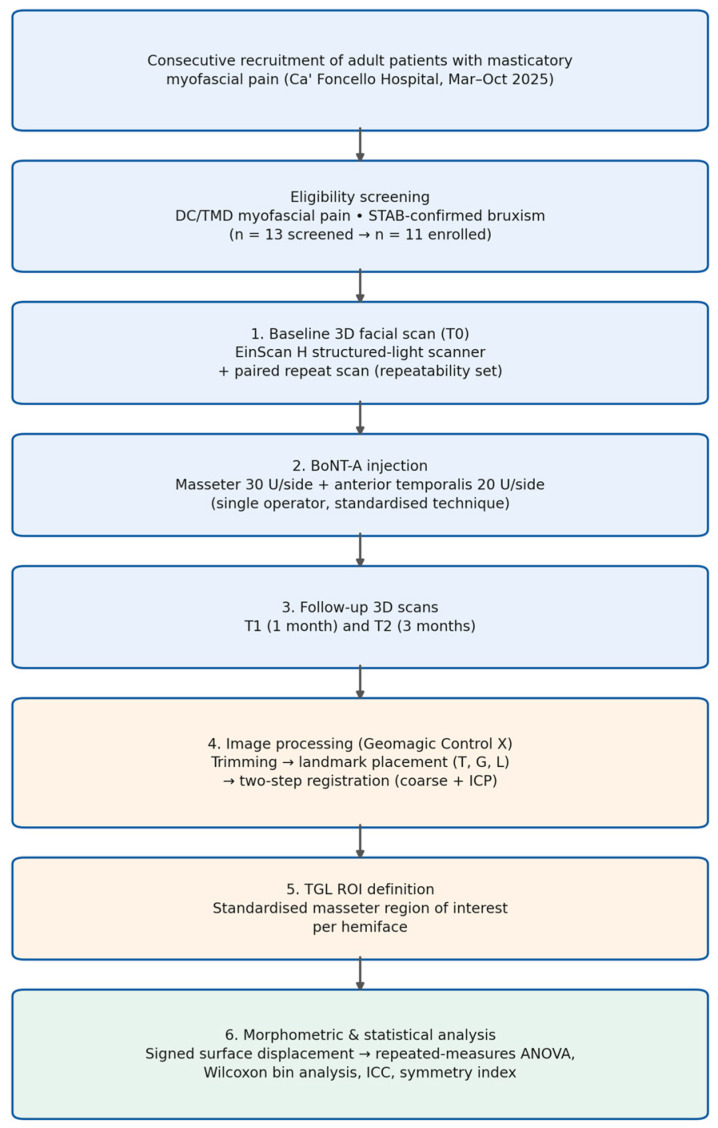
Overview of the study workflow, from patient recruitment to statistical analysis of 3D morphometric outcomes.

Sample size and study design rationale. This investigation was designed as a pilot observational study to estimate the magnitude, variability, and temporal trajectory of 3D morphometric changes following BoNT-A injection, with the goal of informing sample-size planning for future confirmatory trials. No formal sample size calculation was performed a priori; the sample was determined by feasibility and patient availability within the study period. Feasibility objectives included successful acquisition of 3D scans at all timepoints, quantification of measurement repeatability, and completion of follow-up in ≥90% of enrolled participants. Figure 1 summarises the overall study workflow.

### 5.2. Botulinum Toxin Injection Protocol

All participants received a single-session bilateral intramuscular injection of onabotulinumtoxinA (Botox^®^, Allergan/AbbVie, North Chicago, IL, USA) administered by one experienced operator (M.V.) with ≥10 years of clinical experience in orofacial BoNT-A injections, using a standardised technique [20].

Total dose administered: 100 U per patient (50 U per side), reconstituted with sterile 0.9% saline to a concentration of 100 U/mL and injected using 30-gauge needles following standard skin antisepsis and anatomical landmark identification.

Masseter muscle: 30 U per side, distributed across five intramuscular points (6 U per point). Injection sites were positioned over the bulk of the masseter, with medial placement to avoid diffusion into adjacent structures. Anterior temporalis muscle: 20 U per side, distributed across four intramuscular points (5 U per point). This allocation reflects the unit’s standard clinical protocol for diffuse masticatory myofascial pain management [20].

Adverse events and local reactions were documented at each follow-up visit using a standardised checklist (including pain at the injection site, ecchymosis, oedema, dysphagia, asymmetric smile, and other unexpected effects).

### 5.3. Three-Dimensional Facial Scanning and Image Acquisition

Facial 3D scans were acquired at three predetermined timepoints: T0 (baseline, pre-injection), T1 (one month post-injection), and T2 (three months post-injection). All scans were obtained using a structured-light 3D facial scanner (EinScan H, Shining 3D Technology, Hangzhou, China) operated by a trained technician. Standardised acquisition protocol was maintained throughout: seated position, neutral head posture, eyes gently closed, lips relaxed, teeth in light occlusion, consistent ambient lighting (approximately 500–1000 lux), and fixed scanner-to-patient distance (approximately 50 cm). Scanning time per acquisition was approximately 3–5 min per participant. Bite tension was standardised behaviourally rather than mechanically: participants were verbally cued to maintain light habitual tooth contact at every acquisition, and no interocclusal spacer or bite-fork device was used, since such devices can displace peri-oral soft tissue and confound the masseter ROI signal.

Intra-session repeatability assessment. To quantify acquisition-related noise and establish empirical measurement uncertainty, two consecutive 3D scans were acquired at T0 for each participant (*n* = 11 paired scan sets), with brief repositioning between acquisitions. The same registration and ROI pipeline employed for longitudinal comparisons was applied to these paired scans to estimate intra-session repeatability. This approach allowed measurement precision to be characterised independently of treatment-related change.

### 5.4. Three-Dimensional Image Processing, Registration, and ROI Definition

3D surface models were processed using Geomagic Control X software (v2024.0.0, 3D Systems Corp., Rock Hill, SC, USA). Non-relevant anatomical structures (hair, ears where necessary, lower neck, and shoulders) were removed using standardised trimming boundaries defined a priori and applied consistently to all models.

Registration strategy and alignment error quantification. A two-step registration pipeline was employed: (1) coarse alignment based on stable facial anatomical landmarks (nasion, anterior nasal spine, and bilateral tragus); (2) iterative closest point (ICP) refinement restricted to anatomically stable regions (forehead, nasal bridge, and zygomatic area) expected to remain unaffected by BoNT-A injection. The masseter ROI was excluded from the refinement step to avoid circular bias. Registration quality was quantified by the root mean square (RMS) error for each pairwise comparison, reported as mean ± standard deviation across participants.

**Figure 2:**
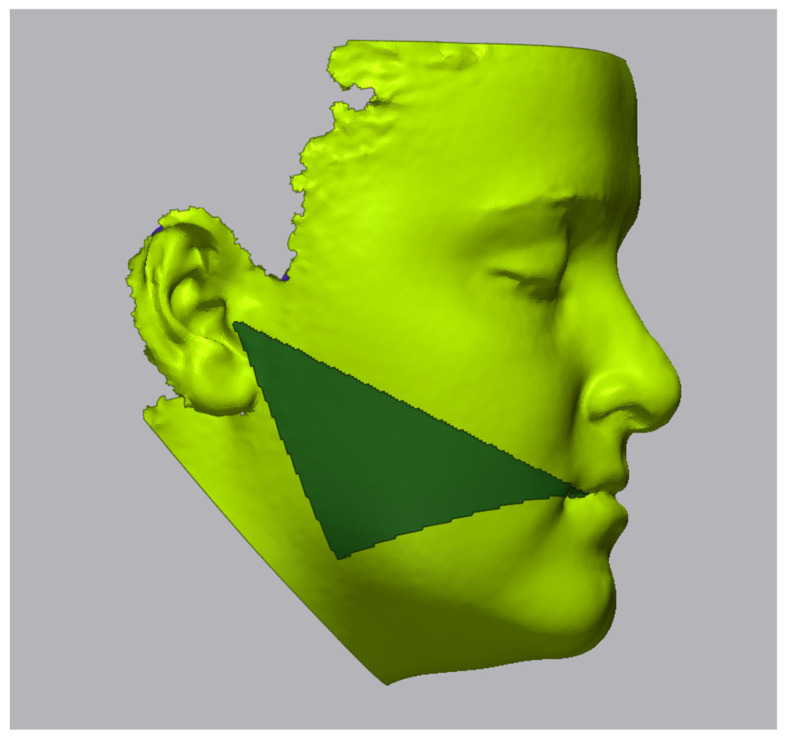
TGL area (Tragus–Gonion–Labial commissure) region of interest definition on 3D facial surface. The three landmark points define the standardised masseter region analysed in the study.

Standardised ROI definition (“TGL area”). A reproducible masseter region of interest for each hemiface was defined using three cutaneous landmarks identified manually on the surface mesh: T (tragus), G (gonion: the most inferolateral point of the mandibular angle), and L (labial commissure). The ROI polygon was generated by connecting these three landmarks and extending boundaries to encompass the superficial and intermediate portions of the masseter muscle (Figure 2). This approach provided a standardised, reproducible, and anatomically relevant definition applicable across all participants and timepoints.

Landmark reliability assessment. Landmarks were placed by a single examiner (L.Z.) blinded to timepoint identifiers. To assess intra-rater reliability, landmark placement was repeated on a random subset of four scans (36% of the cohort) after a two-week washout period. Intraclass correlation coefficients (ICC [3, 1]) with 95% confidence intervals were calculated for each landmark coordinate (x, y, z) on each side using a two-way mixed-effects model for absolute agreement.

### 5.5. Morphometric Outcome Definitions

Surface deviation between registered 3D scans was computed at every point within the masseter ROI, expressed as signed surface displacement (mm). Negative values indicated inward displacement (volumetric reduction), while positive values indicated outward displacement (volumetric expansion). A noise tolerance band of ±0.5 mm was established a priori based on the measured intra-session repeatability of the scanner.

Primary morphometric endpoint: mean signed surface displacement (mm) within the bilateral masseter ROI at T1 and T2 relative to baseline, with standard deviation and 95% confidence intervals.

Secondary morphometric endpoints: (i) unilateral (right and left) mean surface displacement; (ii) proportion of ROI points falling within predefined displacement bins (0 to ±1 mm, −1 to −2 mm, −2 to −3 mm, and corresponding positive bins); (iii) bilateral symmetry index, calculated as the mean absolute difference between left and right ROI mean displacement at each timepoint; (iv) temporal trajectory of recovery between T1 and T2.

### 5.6. Statistical Analysis

Statistical analysis was performed using IBM SPSS Statistics v29.0 (IBM Corp., Armonk, NY, USA) and Python (SciPy v1.11 [43], Pingouin v0.5 [44]). Normality of continuous endpoints was assessed using the Shapiro–Wilk test supplemented by visual inspection of histograms and Q-Q plots.

For the primary endpoint (mean bilateral surface displacement), data at T0, T1, and T2 approximated normality (Shapiro–Wilk *p* ≥ 0.15 at all timepoints). Accordingly, a one-way repeated-measures analysis of variance (ANOVA) was applied, with sphericity correction via the Greenhouse-Geisser epsilon (ε). Effect size was reported as generalised eta-squared (η^2^G). Post hoc pairwise comparisons used paired-samples *t*-tests with Bonferroni correction; effect sizes for pairwise comparisons were expressed as Hedges’ g.

For secondary outcomes expressed as bin-percentages (proportions of surface points within each displacement range), the Friedman test was used across the three timepoints, with post hoc Wilcoxon signed-rank tests (Bonferroni-corrected; α = 0.05/number of comparisons). Effect size for Wilcoxon tests was computed as r = Z/√N. For secondary bin analyses, where only two timepoints were compared, Wilcoxon signed-rank tests were applied directly with effect size r. Because proportion data can violate the assumptions of standard parametric transformations, non-parametric tests were applied directly to the raw bin percentages rather than to arcsine-transformed values [45].

For ICC calculation, a two-way mixed-effects model for absolute agreement was used (ICC [3, 1]). ICC values ≥ 0.90 were considered excellent. Statistical significance was set at *p* < 0.05 throughout.

## Figures and Tables

**Table 1:** Baseline demographic and clinical characteristics (*n* = 11). BMI, body mass index; DC/TMD, Diagnostic Criteria for Temporomandibular Disorders; STAB, Standardised Tool for the Assessment of Bruxism.

Characteristic	Value (*n* = 11)
Age (years), mean ± SD	50.3 ± 15.1
Sex, female/male (*n*)	10/1
BMI (kg/m^2^), mean ± SD	26.4 ± 3.8
Myofascial pain (DC/TMD), *n* (%)	11 (100%)
Bruxism (definite per STAB), *n* (%)	11 (100%)
Prior conservative TMD treatment, *n* (%)	9 (81.8%)
Adverse events (ecchymosis/oedema), *n* (%)	3 (27.3%)
Follow-up completion rate, *n* (%)	11 (100%)

**Table 2:** Intraclass correlation coefficients (ICC [3, 1]) for TGL landmark coordinates (*n* = 4 scans, two-week retest). Values represent ICC estimate with 95% confidence interval.

Landmark	ICC x (95% CI)	ICC y (95% CI)	ICC z (95% CI)	Mean ICC
Tragus R	0.964 [0.891–0.993]	0.978 [0.921–0.996]	0.971 [0.908–0.994]	0.971
Tragus L	0.958 [0.879–0.991]	0.969 [0.906–0.993]	0.963 [0.895–0.992]	0.963
Gonion R	0.941 [0.848–0.987]	0.953 [0.867–0.990]	0.948 [0.859–0.988]	0.947
Gonion L	0.936 [0.840–0.985]	0.944 [0.851–0.988]	0.939 [0.845–0.986]	0.940
Commissure R	0.947 [0.856–0.988]	0.961 [0.884–0.992]	0.955 [0.873–0.990]	0.954
Commissure L	0.942 [0.850–0.987]	0.956 [0.876–0.991]	0.950 [0.863–0.989]	0.949

**Table 3:** Mean signed surface displacement (mm) at each timepoint relative to baseline (T0 = 0 mm by definition). Negative values indicate inward displacement (volumetric reduction). A total of 95% CI, 95% confidence interval; g, Hedges’ g (vs. T0); n.s., not significant.

Comparison	Side	Mean Δ (mm)	SD (mm)	95% CI	p (Bonferroni)	Hedges’ g
T0→T1	Bilateral	−0.716	0.480	−1.000 to −0.433	0.002	2.03
T0→T1	Right	−0.695	0.500	−0.991 to −0.400	0.003	1.93
T0→T1	Left	−0.759	0.505	−1.058 to −0.460	0.002	2.07
T0→T2	Bilateral	−0.847	0.588	−1.195 to −0.500	0.002	1.96
T0→T2	Right	−0.637	0.819	−1.121 to −0.153	0.033	1.08
T0→T2	Left	−0.984	0.449	−1.249 to −0.718	<0.001	2.96
T1→T2	Bilateral	−0.131	0.615	−0.494 to +0.232	1.000	0.23

**Table 4:** Summary of statistically significant displacement bin findings from Wilcoxon signed-rank tests (Bonferroni-corrected). r, effect size r = Z/√N; n.s., not significant (*p* > 0.05).

Comparison	Laterality	Displacement Bin (mm)	W	*p*-Value	r	Interpretation
T0→T1	Bilateral	0 to +1	1.0	0.002	0.93	Shift toward negative values
T0→T1	Bilateral	−1 to −2	3.0	0.005	0.85	Significant increase
T0→T1	Left	−1 to −2	2.0	0.003	0.90	Significant increase
T0→T1	Right	All bins	-	n.s.	-	Directional trend only
T0→T2	Bilateral	−1 to −2	0.0	0.001	0.99	Highly significant increase
T0→T2	Bilateral	−2 to −3	6.0	0.014	0.74	Significant persistent change
T1→T2	All	All bins	-	n.s.	-	No significant bin-level change

## Data Availability

The data presented in this study are available on request from the corresponding authors. The data are not publicly available due to privacy constraints.

## Abbreviations

BoNT-A	Botulinum toxin type A
TMD	Temporomandibular disorder
DC/TMD	Diagnostic Criteria for Temporomandibular Disorders
STAB	Standardised Tool for the Assessment of Bruxism
ROI	Region of interest
ICP	Iterative closest point
RMS	Root mean square
3D	Three-dimensional
EMG	Electromyography
ICC	Intraclass correlation coefficient
STROBE	Strengthening the Reporting of Observational Studies in Epidemiology
